# Orthologous promoters from related methylotrophic yeasts surpass expression of endogenous promoters of *Pichia pastoris*

**DOI:** 10.1186/s13568-020-00972-1

**Published:** 2020-02-25

**Authors:** Thomas Vogl, Jasmin Elgin Fischer, Patrick Hyden, Richard Wasmayer, Lukas Sturmberger, Anton Glieder

**Affiliations:** 1 Institute of Molecular Biotechnology, NAWI Graz, Graz University of Technology, Petersgasse 14, 8010 Graz, Austria; 2 Present Address: Department of Computer Science and Applied Mathematics, Weizmann Institute of Science, 76100 Rehovot, Israel

**Keywords:** *Pichia pastoris*, *Komagataella phaffii*, Orthologous promoters, Methanol-free, Derepression

## Abstract

Methylotrophic yeasts such as *Komagataella phaffii* (syn. *Pichia pastoris*, *Pp*), *Hansenula polymorpha* (*Hp*), *Candida boidinii* (*Cb*) and *Pichia methanolica* (*Pm*) are widely used protein production platforms. Typically, strong, tightly regulated promoters of genes coding for their methanol utilization (MUT) pathways are used to drive heterologous gene expression. Despite highly similar open reading frames in the MUT pathways of the four yeasts, the regulation of the respective promoters varies strongly between species. While most endogenous *Pp* MUT promoters remain tightly repressed after depletion of a repressing carbon, *Hp*, *Cb* and *Pm* MUT promoters are derepressed to up to 70% of methanol induced levels, enabling methanol free production processes in their respective host background. Here, we have tested a series of orthologous promoters from *Hp*, *Cb* and *Pm* in *Pp*. Unexpectedly, when induced with methanol, the promoter of the *HpMOX* gene reached very similar expression levels as the strong methanol, inducible, and most frequently used promoter of the *Pp alcohol oxidase 1* gene (*P*_*PpAOX1*_). The *HpFMD* promoter even surpassed *P*_*PpAOX1*_ up to three-fold, when induced with methanol, and reached under methanol-free/derepressed conditions similar expression as the methanol induced *P*_*PpAOX1*_. These results demonstrate that orthologous promoters from related yeast species can give access to otherwise unobtainable regulatory profiles and may even considerably surpass endogenous promoters in *P. pastoris*.

## Introduction

Recombinant proteins such as biopharmaceuticals or industrially relevant biocatalysts are commonly produced by heterologous gene expression in microorganisms. *Escherichia coli*, *Saccharomyces cerevisiae*, filamentous fungi, and cells of higher eukaryotes have been widely used as expression hosts since the advent of recombinant protein production. Over the past three decades, the methylotrophic yeasts *Pichia pastoris* (*Pp*), *Hansenula polymorpha* (*Hp*), *Candida boidinii* (*Cb*) and *Pichia methanolica* (*Pm*) have emerged as powerful alternatives, enabling high cell density fermentation and simple, pure secretion of heterologous proteins (Gellissen 2000; Hartner and Glieder 2006; Yurimoto et al. 2011; Vogl et al. 2013). The two *Pichia* species and *H. polymorpha* have phylogenetically been reassigned as *Komagataella* and *Ogataea* species, respectively resulting in the formal names *Komagataella phaffii*, *Ogataea polymorpha*, and *Ogataea methanolica* (Peña et al. 2018). Amongst these four methylotrophic yeasts, *P. pastoris* is most commonly applied for heterologous protein production, even surpassing *S. cerevisiae* according to a recent literature survey (Bill 2014).

All methylotrophic yeasts offer tightly regulated, strong promoters that are naturally regulating the expression of genes involved in the methanol utilization (MUT) pathway (Hartner and Glieder 2006). Typically, all promoters of MUT genes are tightly repressed on repressing carbon sources such as glucose and get strongly upregulated when shifted to methanol. However, derepression effects vary considerably between species (Hartner and Glieder 2006) and even within the same organism (Vogl et al. 2016). Derepression leads to activation of the promoter when the repressing carbon source is depleted or when a non-repressing carbon source is present. Under derepressed conditions, the promoter of the *alcohol oxidase 1* gene in *P. pastoris* (*P*_*PpAOX1*_) is only activated at 2–4% compared to methanol induced levels (Vogl and Glieder 2013). Although some of the MUT promoters of *P. pastoris* showed substantial derepression effects, their efficiency was considerably lower than methanol induced *AOX1*, *DAS1* or *DAS2* promoters (Vogl et al. 2016). In contrast, the promoter of the orthologous gene (named differently: *methanol oxidase*, *MOX*) in *H. polymorpha* (*P*_*HpMOX*_) shows derepressed expression up to 70% of methanol induced levels, even in presence of glycerol whereas *P*_*PpAOX1*_ is fully repressed by glycerol. Also the promoters of the orthologous genes in *C. boidinii* (*alcohol oxidase 1*, abbreviated *AOD1*) and *P. methanolica* (*methanol oxidase 1/2*, abbreviated *MOD1/2*) were reported to be activated by derepression, reaching up to 70% of methanol induced levels (Hartner and Glieder 2006). However, the use of the orthologous *AOX1* promoter of *Pp* in *Hp* indicated that the respective regulation is host specific rather than due to the specific promoter sequence since glycerol did not repress the *P*_*PpAOX1*_ in *Hp* (Rodriguez et al. 1996; Raschke et al. 1996). Note that the *alcohol oxidase*/*methanol oxidase* genes fulfilling the same function were assigned different three letter abbreviations in all four yeasts. We are keeping these identifiers in addition to the prefixes *Pp*, *Hp*, *Cb* and *Pm* to differentiate between the organisms.

Especially in large scale production processes and for biopharmaceutical production, induction with toxic and flammable methanol is unwanted due to safety issues, making strong derepressed promoters sought-after expression tools to enable methanol free processes. Derepressed promoters allow for regulated expression by simply varying the availability of the carbon source [i.e. repression is achieved with an excess of a repressing carbon source, subsequently reducing the feed rate to limiting amounts triggers activation e.g. (Hartner et al. 2008; Vogl et al. 2018c)]. *P*_*PpAOX1*_ variants (Hartner et al. 2008), alternative promoters (Prielhofer et al. 2013), novel MUT promoters (Vogl et al. 2016), synthetic bidirectional promoters (Vogl et al. 2018b) and altering the molecular regulation of *P*_*PpAOX1*_ (Shen et al. 2016a, b; Wang et al. 2017; Vogl et al. 2018c) showed derepression to varying extents in *P. pastoris*.

Recent studies in metazoans (Weirauch and Hughes 2010) and yeast (Zeevi et al. 2014) have shown that orthologous, highly divergent promoter sequences from different species can achieve similar expression. For example, the promoters of the genes coding for orthologous ribosomal proteins in various yeast species, showed high expression conservation in *S. cerevisiae* (Zeevi et al. 2014). We hypothesized that also MUT promoters of related methylotrophic yeasts may show some extent of conservation. Here we have tested a comprehensive series of commonly used MUT promoters from *Hp*, *Cb* and *Pm* in *Pp* and some of these promoters performed surprisingly well, even outperforming the most frequently used endogenous *Pp* promoters.

## Materials and methods

### Cloning of promoters

The orthologous promoters were PCR amplified and cloned upstream of an eGFP reporter gene into a previously established reporter plasmid for *P. pastoris* [pPpT4mutZeoMlyI-intARG4-eGFP-BmrIstuffer, (Vogl et al. 2016)] based on the pPpT4 vector reported by Näätsaari et al. (2012). The promoters were cloned seamlessly, i.e. maintaining the natural sequence context to the start codon without additional restriction endonuclease sites or linker sequences. Primers were designed according to the literature (*HpFMD/MOX* promoters (Ledeboer et al. 1985; Song et al. 2003), *CbAOD1* [Yurimoto et al. 2000) and *CbFLD1* (Lee et al. 2002), *Pm MOD1* and *MOD2* (Raymond et al. 1998; Nakagawa et al. 2001, 2006)] and the primer sequences are provided in Additional file 1: S1. Genomic DNA of the strains *Hp* DSM 70277, *Cb* DSM 70026 and *Pm* DSM 2147 was isolated and used as template for the PCR reactions. The PCRs were cloned into the reporter vector by TA cloning as outlined previously (Vogl et al. 2015, 2016). The cloned promoters were verified by Sanger sequencing, showing in part minor differences to previously reported sequences (Additional file 1: S2). The control vectors of the *P. pastoris* endogenous *AOX1*, *CAT1* and *GAP* promoters were available from previous studies (Vogl et al. 2016).

The alternative reporter vectors bearing HRP [isoenzyme A2A (Näätsaari et al. 2014)], CalB and *Me*HNL downstream of the respective promoters were in part available from previous studies (Vogl et al. 2016) or generated by cutting out the eGFP reporter gene from the above mentioned vectors (via NheI and NotI restriction sites) and seamlessly inserting PCR products of the GOIs by assembly cloning (Gibson et al. 2009). See Additional file 1: S1 for the primer sequences and Additional file 1: S4 for a list of the plasmids and strains used in this study. The HRP and CalB vectors previously reported (Vogl et al. 2016) were used as PCR templates, the *Me*HNL sequence was codon optimized for *P. pastoris* and ordered with overhangs to the *AOX1* promoter and terminator for assembly cloning (Additional file 1: S1). This vector was sequenced and used as template for PCR amplification. Since the HRP and CalB genes were both fused to a mating factor alpha signal sequence, the same forward primer could be used for amplification (pHpFMD-MFalpha-Gib). The inserted genes were sequenced with primers binding to the *AOX1* terminator and the respective promoters (Vogl et al. 2016), for *P*_*HpFMD*_ primer seq-pHpHMD-149..126fwd was used to allow a new Sanger sequencing of the downstream gene.

### Strains, materials, fluorescence measurements and enzyme assays

Materials and strains were used as previously reported in detail (Vogl et al. 2016). Deep well plate and shake flask cultivations were also performed as reported in the literature (Weis et al. 2004; Vogl et al. 2016). Fluorescence measurements, HRP and CalB activity assays were also performed as previously reported (Vogl et al. 2016). Culture supernatants for the HRP and CalB activity assays were obtained by centrifugation (3000*g* for 20 min) and carefully transferring the liquid without touching the pelleted cells. For MeHNL activity measurements, cell free extracts were generated in fourfold replicates from independently grown cultivations of the same strain (Vogl et al. 2018c) by centrifugation (3000*g* for 20 min), resuspending the pellet in 200 µL Y-PER (Thermo Scientific), shaking for 30 min followed by 30 min (3000*g*) centrifugation. The resulting supernatant was typically diluted at least tenfold for the MeHNL activity measurement [as described in (Hanefeld et al. 1999) using a mandelonitrile cyanogenesis assay (Wiedner et al. 2014) with a final mandelonitrile concentration of 15 mM]. For transformations of all basic promoter comparisons, the *P. pastoris* CBS7435 wildtype strain was used following the condensed protocol of Lin-Cereghino et al. (2005), see the following section for applied DNA amounts and the screening/rescreening procedure of transformants. Plasmids were linearized with SwaI prior to transformation (Vogl et al. 2018a). During transformation and selection of *P. pastoris*, we noticed for the *CbAOD1* promoter transformation background (colonies showing no reporter protein expression when re-cultivated), as previously noticed for extended lengths of the *P. pastoris CAT1* promoter (Vogl et al. 2016). As *P*_*CbAOD1*_ did not show any reporter protein fluorescence, we did not further investigate this phenomenon during this study. HRP and CalB were used for transformation of a mutS (methanol utilization slow, *Δaox1*) strain, as higher yields have been reported (Krainer et al. 2012) and the muts strain was also used for the control plasmids bearing these genes of interest under the control of *P. pastoris* endogenous promoters (Vogl et al. 2016).

### Screening, rescreening procedures and culture conditions

To avoid clonal variation due to different copy numbers of integrated expression cassettes as well as different integration sites and genomic alterations that can bias expression strength comparisons in *P. pastoris* (Schwarzhans et al. 2016a, b; Vogl et al. 2018a), transformants from this study underwent the following screening and rescreening procedures [the section is adapted from the open access publication (Vogl et al. 2018b)]. *P. pastoris* cells were transformed with molar equivalents to 1 µg of the empty pPpT4_S vector SwaI linearized plasmids as 1 µg of the empty pPpT4_S vector was found to yield predominantly single copy integration (Vogl et al. 2014, 2018a). The screening and rescreening procedures to compare single *P. pastoris* strains have previously been reported (Vogl et al. 2014, 2016, 2018b) in detail. In brief, for each construct 42 transformants (approximately half a DWP) were screened to avoid clonal variation observed in *P. pastoris* (Schwarzhans et al. 2016a, 2016b; Vogl et al. 2018a). Four representative clones from the middle of the obtained expression landscape were streaked for single colonies and rescreened in biological 7-fold replicates (raw data provided as Additional file 1: S3) to avoid outliers of multi-copy integration or reduced expression because of deletions or undesired integration events (Schwarzhans et al. 2016a, b; Vogl et al. 2018a) were streaked for single colonies and rescreened in biological sevenfold replicates. Finally, one representative clone was selected and a final screening of all the promoters together was performed (data shown in the figures of the main manuscript). *P. pastoris* strains were grown for 60 h on 250 µL BMD1 media (buffered minimal dextrose with 1% glucose) and subsequently induced with methanol (250 µL BMM2 [1% methanol] at 60 h and 50 µL BMM10 [5% methanol] at 72 h followed by intervals of 24 h if applicable). Inoculation was performed with ~ 10 µL of frozen glycerol stocks (equaling to an approx. initial OD < 0.05). The BMD and BMM minimal media contain 0.2 M/L potassium phosphate buffer (pH 6), 13.4 g/L yeast nitrogen base and 0.4 mg/L biotin and only differ in the carbon source (as indicated above).

### Accession numbers

Orthologous promoters (GenBank): MA887959, MA887960, MA887981, MA887982, MA887983, MA887984; Codon-optimized MeHNL gene: MA887980.

## Results

### Comparison of orthologous yeast promoters in *P. pastoris*

Based on their known high promoter activity and their frequent use in their native host (Hartner and Glieder 2006), we selected six orthologous promoters of the *HpFMD*, *HpMOX*, *CbFLD1*, *CbAOD1*, *PmMOD1* and *PmMOD2* genes for functional evaluation in *P. pastoris* (Table 1). These promoters have been reported to be amongst the strongest methanol inducible promoters and at the same time the most derepressed promoters in the respective organisms [reviewed by (Hartner and Glieder 2006)]. These promoters were compared to state of the art endogenous promoters which were so far most frequently used in *P. pastoris*, i.e. the methanol inducible *P*_*AOX1*_, constitutive *P*_*GAP*_, and derepressed/methanol inducible *P*_*CAT1*_ (Vogl et al. 2016) (Table 1). The orthologous promoters were PCR amplified from genomic DNA and cloned into a reporter vector previously established for promoter comparisons in *P. pastoris* (Vogl et al. 2016). The promoters were seamlessly fused (i.e. maintaining the natural transition of promoter to start codon without additional restriction sites or linker sequences in between) to an enhanced green fluorescent reporter gene (eGFP). DNA sequencing showed that the promoter sequences contained minor differences compared to previous reports (Additional file 1: S2). These differences are possibly arising from the use of genomic DNA from *Hp*, *Cb* and *Pm* strains from different strain collections than previously reported as PCR templates (see “Materials and methods” section).

**Table 1:** Orthologous MUT promoters of related species and endogenous *P. pastoris* promoters used in this study

Type	Abbreviation	Species	Gene name	Regulation in native species	Length (bp)	GC content (%)
Orthologous promoters	*HpFMD*	*Hansenula polymorpha*	Formate dehydrogenase	Strongly derepressed, methanol inducible	623	53.3
	*HpMOX*	*Hansenula polymorpha*	Methanol oxidase	Strongly derepressed, methanol inducible	1510	56.0
	*CbFLD1*	*Candida boidinii*	Formaldehyde dehydrogenase	Moderately derepressed, methanol inducible	572	31.6
	*CbAOD1*	*Candida boidinii*	Alcohol oxidase 1	Moderately derepressed, methanol inducible	1652	28.6
	*PmMOD1*	*Pichia methanolica*	Methanol oxidase 1	Strongly derepressed, methanol inducible	1157	37.9
	*PmMOD2*	*Pichia methanolica*	Methanol oxidase 2	Tightly repressed, methanol inducible	1662	37.3
*P. pastoris* endogenous promoters	*PpAOX1*	*Pichia pastoris*	Alcohol oxidase 1	Tightly repressed, methanol inducible	940	42.6
	*PpCAT1*	*Pichia pastoris*	Catalase 1	Moderately derepressed, methanol inducible	500	40.8
	*PpGAP*	*Pichia pastoris*	Glyceraldehyde 3-phosphate dehydrogenase	Constitutive	486	46.7

Moderately derepressed: < 50% of methanol induced levels; strongly derepressed: > 50% of methanol induced levels [according to the data by (Hartner and Glieder 2006)]. Promoter lengths used in this study are listed and deviate in part slightly form values reported in the literature (see “Materials and methods” section and Additional file 1: S2)

### The *HpFMD* promoter enables strong derepressed expression in *P. pastoris*

**Fig. 1:**
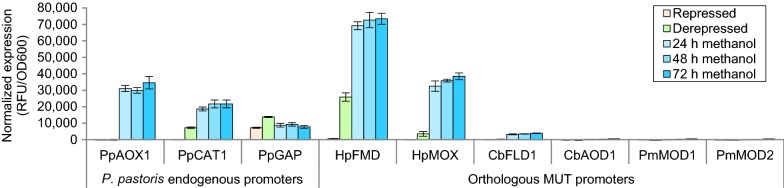
Orthologous MUT promoters outperform *P. pastoris* endogenous promoters. Reporter protein fluorescence of all orthologous and *P. pastoris* endogenous promoters tested. The orthologous MUT promoters of different methylotrophic yeasts were cloned upstream of an enhanced green fluorescent protein (eGFP) and transformed into *P. pastoris*. The strains were cultivated in deep well plates (DWPs) on BMD1 (glucose) media and subsequently induced with methanol (Weis et al. 2004; Vogl et al. 2016). Reporter protein fluorescence and OD_600_ were measured under glucose repressed (16 h) and derepressed (60 h) conditions and different time points of methanol induction. Fluorescence measurements were normalized per OD_600_. Mean values (MVs) and standard deviations (SDs) of biological quadruplicates are shown

*Pichia pastoris* transformants of plasmids bearing *CbAOD1*, *PmMOD1* and *PmMOD2* promoters did not show any reporter protein fluorescence (Fig. 1). *P*_*CbFLD1*_ showed repression on glucose and weak methanol inducible expression of about 10% of *P*_*PpAOX1*_, in line with the initial expectation that host specific regulatory proteins and mechanisms are necessary for efficient transcription. However, both *H. polymorpha* promoters tested unexpectedly maintained their natural regulation and showed repression, derepression and methanol induction profiles. The *HpMOX* promoter showed weak derepressed reporter protein fluorescence and reached similar reporter protein fluorescence on methanol as *P*_*PpAOX1*_. The *HpFMD* promoter showed derepressed expression outperforming the constitutive *P*_*PpGAP*_ and reaching approximately 75% of the methanol induced *P*_*PpAOX1*_ for the well expressible intracellular eGFP reporter. Derepressed expression from *P*_*HpFMD*_ exceeded reporter protein fluorescence of the strongest derepressed endogenous MUT promoter from *P. pastoris* (*P*_*PpCAT1*_) considerably and upon methanol induction *P*_*HpFMD*_ even outperformed *P*_*PpAOX1*_ ca. 2.1-fold.

**Fig. 2:**
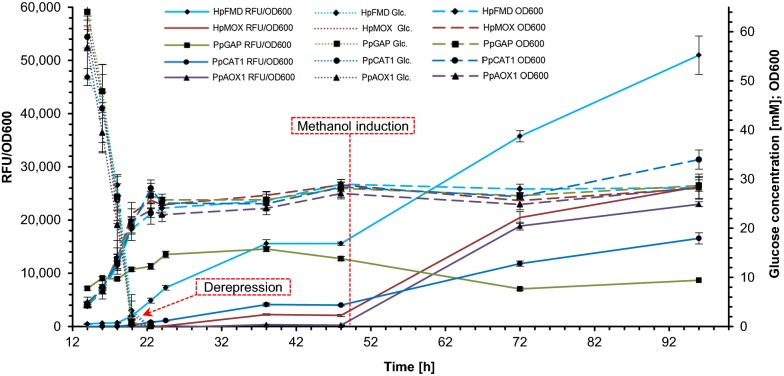
*P*_*HpFMD*_ enables strong derepression and exceeds the strength of methanol induced endogenous *P. pastoris* promoters. Strains bearing selected promoters from Fig. 1 (*HpFMD*, *HpMOX*, *PpAOX1*, *PpCAT1*, *PpGAP*) were cultivated in shake flasks and inoculated to a low starting OD_600_ of 0.05. Reporter protein fluorescence, OD_600_ and glucose levels were measured. Fluorescence/OD_600_ values at t = 0 are not shown, as the starting OD_600_ of 0.05 was outside the linear range of the spectrometer used. The initial glucose concentration of the media was 55.5 mM (10 g/L). MVs and SDs of biological triplicates are shown

In deep well plate cultivations (Fig. 1) *P*_*HpFMD*_ seemed to give also a very weak reporter fluorescence signal under glucose repressed conditions, hinting slight constitutive activity. Expression from the *P*_*HpMOX*_ and *P. pastoris P*_*AOX1*_ and *P*_*CAT1*_ was undetectable. In experiments in shake flasks measuring also glucose levels (Fig. 2), *P*_*HpFMD*_ showed very weak constitutive expression before full depletion of glucose. This result may suggest that the exceptional strength of *P*_*HpFMD*_, clearly outperforming even *P. pastoris* endogenous promoters, is at the expense of less tight repression at lower glucose concentrations. Constitutive activity of *P*_*HpFMD*_ is less than 1% of fully induced levels, showing still induction over two logs.

### Validation of *P*_*HpFMD*_ promoter with additional reporter genes

**Fig. 3:**
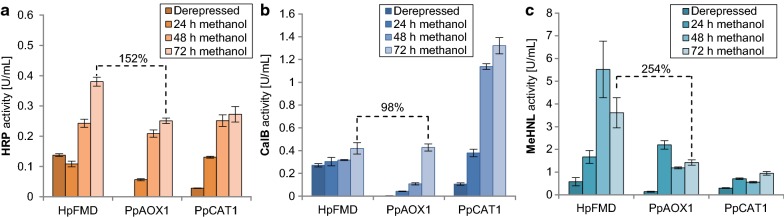
Applying the *HpFMD* promoter for expression of the enzymes HRP (**a**), CalB (**b**) and MeHNL (**c**) confirms the high expression observed with eGFP (Fig. 1). The strains were grown in DWPs on BMD1 media until glucose depletion for 60 h and were subsequently induced with methanol. HRP and CalB activities in the supernatants were measured and cells lysed to measure intracellular MeHNL activity. Mean values (MVs) and standard deviation (SDs) of biological quadruplicates are shown. The activities of the methanol induced *HpFMD* promoter compared to the state of the art *AOX1* promoter are highlighted (after 72 h of methanol induction)

Since strong transcription not always favors expression of other proteins, especially when secreted, we were interested if the exceptionally strong expression of *P*_*HpFMD*_ could also be reproduced with other proteins than eGFP. Therefore, the *P*_*HpFMD*_ promoter was cloned upstream of the coding sequence of secretory proteins horseradish peroxidase (HRP) and *Candida antarctica* lipase B (CalB) and an intracellularly expressed hydroxynitrile lyase from *Manihot esculenta* (*Me*HNL) (Fig. 3). Yields obtained from *P*_*HpFMD*_ were compared to the *P. pastoris* endogenous MUT promoters *P*_*PpCAT1*_ and *P*_*PpAOX1*_. Derepressed expression of HRP and CalB employing *P*_*HpFMD*_ clearly outperformed derepressed expression from *P*_*PpCAT1*_. Methanol induced enzyme activities of *P*_*PpCAT1*_ and *P*_*PpAOX1*_ were similar, only for CalB expression *P*_*PpCAT1*_ outperformed all tested promoters, suggesting a specific beneficial effect. Methanol induced activities from *P*_*HpFMD*_ outperformed methanol induced *P*_*PpAOX1*_ up to 2.5-fold. However, the effect was stronger for the intracellular expression of *MeHNL* (Fig. 3c) than the secretory expression of HRP and CalB (Fig. 3a, b). We assume that for the secretory proteins, not transcription but rather passage through the secretory pathway is the limiting factor. In line with this hypothesis, it has previously been shown that multicopy strains of CalB even show reduced activities compared to single copy if expressed without helper proteins (Abad et al. 2010). Similar effects were also noticed for HRP (Krainer et al. 2016), where maximum titers obtained so far are still in the several 100 mg/L range. Too strong overexpression of HRP and CalB by *P*_*HpFMD*_ may overburden the secretion machinery [‘secretion saturation’ (Aw and Polizzi 2013)] or other factors such as cofactor synthesis might limit product titers, whereas intracellular expression of *Me*HNL appears more simple and well tolerated by the host.

The strong expression from *P*_*HpFMD*_ was consistently reproducible using four reporter genes (eGFP, HRP, CalB, *Me*HNL), demonstrating that orthologous promoters from related organisms can be valuable tools for protein production even exceeding endogenous promoters.

## Discussion

Here we have shown that orthologous MUT promoters can be highly useful tools for single protein production, as demonstrated by up to 3.5-fold higher expression achieved form the *P*_*HpFMD*_ compared to the strongest endogenous *P. pastoris* MUT promoters. Interestingly, although regulating genes of proteins with high sequence similarity and similar enzymatic function, the respective orthologous promoters show highly divergent sequences from *P. pastoris*. None of the orthologous promoters tested show clear identity to the *P. pastoris* genome when performing a BLAST search (using standard parameters) and also alignments to their *P. pastoris* orthologs did not exhibit clear identities (data not shown). A similar lack of sequence identity between similarly regulated promoters was reported for *P. pastoris* MUT promoters (Vogl et al. 2016), in metazoans (Weirauch and Hughes 2010) and *S. cerevisiae* (Zeevi et al. 2014). We assume that the expression from these MUT promoters is governed by short, partially degenerative transcription factor binding sites (TFBS), also conserved in some orthologous promoters. The *P. pastoris* methanol master regulator Mxr1p (Lin-Cereghino et al. 2006) binds for example a simple CYCCNY motif and this motif is dispersed over different positions in the *P. pastoris AOX1*, *DAS2* and *PEX8* promoter sequences (Kranthi et al. 2009, 2010).

Such high sequence diversity and lack of identity is especially advantageous if multiple genes should be co-expressed. The repeated use of identical sequences can results in ‘loop out’ recombination in yeast (Aw and Polizzi 2013), leading to loss of copies or parts of expression cassettes (Zhu et al. 2009; Geier et al. 2015; Schwarzhans et al. 2016a, b; Vogl et al. 2018a). To this end, orthologous promoters with similar regulation but dissimilar sequences may also become valuable tools for metabolic engineering and synthetic biology endeavors, requiring the expression of multiple genes from similarly regulated promoters (Vogl et al. 2016).

The strong derepressed activity of the *HpFMD* promoter is even more surprising in view of this large sequence diversity. It has previously been suggested, that regulation of derepression in methylotrophic yeasts is conferred primarily by the host regulatory machinery and not by the promoter sequences (Hartner and Glieder 2006). This assumption was taken, as the *P. pastoris AOX1* promoter (tightly repressed in its natural host) did not maintain its tight repression if transferred to *H. polymorpha. P*_*PpAOX1*_ showed in *Hp* derepression similar to endogenous *H. polymorpha* promoters (Rodriguez et al. 1996; Raschke et al. 1996; Hartner and Glieder 2006). However, in our hands the *HpFMD* promoter exhibited strong derepressed expression in *P. pastoris*, unlike strong *P. pastoris* endogenous promoters. A possible explanation may be that *P. pastoris* contains unique repressors to maintain tight repression under derepressed conditions. It appears that this machinery does not exist in *H. polymorpha* [or at least does not act on the *HpFMD* and *HpMOX* promoters, as these promoters are naturally derepressed and also the *PpAOX1* promoter is derepressed in presence of glycerol when applied in *Hp* (Raschke et al. 1996)]. So it is unlikely that the *HpFMD* and *HpMOX* promoters contain binding sites for the *P. pastoris* machinery to maintain tight repression, which would explain their derepressed regulation in *P. pastoris*. Alternatively, the effect may also be explained by an activating model: *H. polymorpha* may contain activators that start expression under derepressed conditions. *P. pastoris* may contain similar derepressed activators, as for example the *PpCAT1* promoter is also moderately derepressed (Vogl et al. 2016). The *HpFMD* promoter may contain more TFBS for these activators than *P*_*PpCAT1*_, leading to stronger activation. However, these are just hypotheses and elucidating the exact mechanisms of the strong derepressed expression will require further studies.

Especially the use of the *HpFMD* promoter enables strong expression without employing methanol and provides several advantages over alternative strategies to achieve methanol-free, regulated expression in *P. pastoris.* Due to its sheer strength, *P*_*HpFMD*_ surpasses under methanol-free conditions derepressed *P*_*PpAOX1*_ variants (Hartner et al. 2008) and naturally derepressed promoters such as *P*_*PpCAT1*_ (Vogl et al. 2016). In contrast to approaches of achieving derepressed expression from *P*_*PpAOX1*_ by overexpression of transcription factors or knockout of repressors (Shen et al. 2016a, b; Wang et al. 2017; Vogl et al. 2018c), the use of *P*_*HpFMD*_ does not require genetic modifications of the production strains and can be readily applied in unmodified wildtype strains. However, transcription factor overexpression (Vogl et al. 2018c) does provide an advantage, as thereby existing high level production strains can be easily retrofitted for methanol free production. Despite the establishment of improved genome editing tools for *P. pastoris* (Weninger et al. 2015, 2016, 2018; Raschmanová et al. 2018), it is considerably more difficult to replace the promoters in existing strains. For the generation of novel expression strains the use of *P*_*HpFMD*_ appears favorable and may even be boosted by molecular regulation alterations demonstrated for *P*_*PpAOX1*_ (Shen et al. 2016a, b; Wang et al. 2017; Vogl et al. 2018c). Also, the different strength of expression of *P*_*HpFMD*_ under derepressed and methanol induced conditions also allow consecutive induction which might a reasonable explanation why final yields and titers are seemingly higher with such 2-step induction procedures for non-trivial secreted proteins such as CalB, where strong promoters or multicopy integration of expression cassettes are usually counteracting high titers of folded and active secreted product.

Eventually, *P*_*HpFMD*_ can also be induced with methanol, if the derepressed yields should be exceeded and methanol induction is feasible. In this setup, it represents to the best of our knowledge the strongest promoter reported in *P. pastoris* so far, exceeding the state of the art *AOX1* promoter up to three-fold. In a broader view, our work demonstrates that orthologous promoters from related yeast species can give access to otherwise unobtainable regulatory profiles and may even considerably surpass endogenous promoters, suggesting that this strategy may also be generalized for the discovery of potent, orthologous promoters in other eukaryotic hosts.

## Supplementary information

**Additional file 1.** Supporting information including S1 (primer sequences), S2 (promoter alignments), S3 (screening data), and S4 (list of plasmids and strains).

## Data Availability

References for all DNA sequences used are provided, for the primary promoter sequences changes to previously reported sequences are provided in Additional file.
