# Supplementary material for: Orthologous promoters from related methylotrophic yeasts surpass expression of endogenous promoters of Pichia pastoris
Source: AMB Express. 2020 Feb 25;10:38. doi: 10.1186/s13568-020-00972-1 (PMC7042429; doi:10.1186/s13568-020-00972-1)
Supplement: Supplementary file 1 — Additional file 1. Supporting information including S1 (primer sequences), S2 (promoter alignments), S3 (screening data), and S4 (list of plasmids and strains). [file 13568_2020_972_MOESM1_ESM.docx]

**Additional file 1**

## Journal name

AMB Express

## Title

Orthologous promoters from related methylotrophic yeasts surpass expression of endogenous promoters of *Pichia pastoris*

## Authors

Thomas Vogl^1~^, Jasmin Elgin Fischer^1#^, Patrick Hyden^1#^, Richard Wasmayer^1#^, Lukas Sturmberger^1^, Anton Glieder^1^*

^#^ These authors contributed equally.

*Corresponding author (a.glieder@tugraz.at)

^1^ Institute of Molecular Biotechnology, NAWI Graz, Graz University of Technology, Petersgasse 14, Graz 8010, Austria

ORCIDs: TV: 0000-0002-3892-1740

^~^ Current address: Department of Computer Science and Applied Mathematics, Weizmann Institute of Science, Rehovot 76100, Israel

## S 1 - Primer sequences and synthetic *MeHNL* gene

S 1: Primer sequences and synthetic MeHNL gene

Primers for amplification of the orthologous promoters:

| HpFMDfwd | AATGTATCTAAACGCAAACTCCGAGCTG |
| --- | --- |
| HpFMDrev | GATTTGATTGATGAAGGCAGAGAGCGCAAG |
| HpMOXfwd | TCGACGCGGAGAACGATCTCCTCGAGCT |
| HpMOXrev | TTTGTTTTTGTACTTTAGATTGATGTCACCACCGTGCACTGGCAG |
| PmMOD1fwd | CGAGATGGTACATACTTAAAAGCTGCCATATTGAG |
| PmMOD1rev | TTTGAGAAATTAATAGTAAGATTTTTTTTTCGTAAAAGTTTTGATTGAGTTAATTC |
| PmMOD2fwd | GGATCCACTACAGTTTACCAATTGATTACGCCAATAG |
| PmMOD2rev | TTTGAATTTTAGTTTTAGATAGATAAATATAATTTTCAATCCTGTTATAAAATAGTATAT |
| CbAOD1fwd | GGAGTATACGTAAATATATAATTATATATAATCATATATATGAATACAATGAAAG |
| CbAOD1rev | TATTGAAAAATAATTTTGTTTTTTTTTTTTTGTTTTTTTAAAAGTTCGTTAAAATTCG |
| CbFLD1fwd | GGATCCCTTCAACAGCGGAGTCTCAAAC |
| CbFLD1rev | TTTTGTGGAATAAAAAATAGATAAATATGATTTAGTGTAGTTGATTCAATCAATTGAC |

Primers for cloning the promoters upstream of different reporter genes:

| pHpFMD-MFalpha-Gib | cttgcgctctctgccttcatcaatcaaatcATGAGATTCCCATCTATTTTCACCGCTGTC |
| --- | --- |
| AOX1TT-NotI-CalB | caaatggcattctgacatcctcttgaGCGGCCGCttatggggtcacgataccggaacaag |
| AOX1TT-NotI-HRPA2A | caaatggcattctgacatcctcttgaGCGGCCGCttaggatccgttaactttcttgcaatcaagtc |
| seq-pHpHMD-149..126fwd | actggtgtccgccaataagaggag |
| pHpFMD-MeHNL | cttgcgctctctgccttcatcaatcaaatcATGGTTACTGCTCACTTCGTCTTGATTCAC |
| AOX1TT-NotI-MeHNL | caaatggcattctgacatcctcttgaGCGGCCGCTTAAGCGTAAGCGTCGGCAACTTCCTG |
| pCAT1-MeHNL-Gib | cacttgctctagtcaagacttacaattaaaATGGTTACTGCTCACTTCGTCTTGATTCAC |

Codon optimized *MeHNL* gene:

The CDS is written in upper case, start and stop codons written in bold, overhangs for Gibson assembly (Gibson et al. 2009) to the entry vector are written in lower case, *EcoR*I and *Not*I restriction sites typically used for cloning in the pPpT4 vector family are underlined.

cgacaacttgagaagatcaaaaaacaactaattattgaaagaattccgaaacg**ATG**GTTACTGCTCACTTCGTCTTGATTCACACTATCTGTCATGGTGCTTGGATCTGGCACAAGTTGAAGCCAGCATTGGAGAGAGCTGGACATAAGGTTACCGCTCTTGATATGGCTGCATCTGGTATTGATCCTCGTCAAATCGAACAAATCAATTCATTCGACGAGTACTCAGAGCCACTGCTGACCTTCTTGGAAAAGTTGCCTCAAGGTGAAAAGGTGATCATCGTTGGTGAATCCTGTGCTGGATTGAACATTGCCATTGCAGCTGATAGATATGTCGATAAGATCGCTGCTGGTGTCTTCCACAACTCTCTGTTACCAGATACTGTTCACTCTCCATCTTACACTGTCGAGAAGTTGTTAGAATCATTCCCAGATTGGAGAGATACTGAATACTTTACTTTCACTAACATCACTGGAGAGACTATCACCACCATGAAACTTGGATTCGTTTTGTTGAGAGAAAACCTTTTCACCAAGTGTACTGATGGTGAATACGAATTGGCCAAGATGGTTATGAGAAAGGGTTCTTTGTTTCAGAATGTTCTTGCACAAAGACCAAAGTTCACCGAAAAGGGTTACGGTTCTATCAAGAAGGTCTACATCTGGACTGATCAGGACAAGATCTTCCTGCCAGACTTCCAAAGATGGCAAATCGCAAACTACAAACCAGATAAGGTCTACCAAGTCCAAGGTGGTGATCACAAGTTACAATTGACCAAGACCGAAGAGGTCGCTCACATCTTGCAGGAAGTTGCCGACGCTTACGCT**TAA**gcggccgctcaagaggatgtcagaatgccatttgcctg

## S 2 - Alignments of promoter sequences to references

S 2: The sequences of the orthologous promoters obtained in this study show minor differences to sequences reported in literature. Promoter sequences reported in the literature (denoted as ‘ref’, see materials and methods section) are compared to sequences obtained by PCR amplification and cloning in this study (denoted by an asterisk ‘*’). The lengths reported in the literature up to the start codon are shown.

### HpFMD

HpFMDref AATGTATCTAAACGCAAACTCCGAGCTGGAAAAATGTTACCGGCGATGCGCGGACAATTT 60

HpFMD* AATGTATCTAAACGCAAACTCCGAGCTGGAAAAATGTTACCGGCGATGCGCGGACAATTT 60

************************************************************

HpFMDref AGAGGCGGCGAATCAAGAAACACCTGCTGGGCGAGCAGTCTGGAGCACAGTCTTCGATGG 120

HpFMD* AGAGGCGGCGA-TCAAGAAACACCTGCTGGGCGAGCAGTCTGGAGCACAGTCTTCGATGG 119

*********** ************************************************

HpFMDref GCCCGAGATCCCACCGCGTTCCTGGGTACCGGGACGTGAGGCAGCGCGACATCCATCAAA 180

HpFMD* GCCCGAGATCCCACCGCGTTCCTGGGTACCGGGACGTGAGGCAGCGCGACATCCATCAAA 179

************************************************************

HpFMDref TATACCAGGCGCCAACCGAGTCTCTCGGAAAACAGCTTCTGGATATCTTCCGCTGGCGGC 240

HpFMD* TATACCAGGCGCCAACCGAGTCTCTCGGAAAACAGCTTCTGGATATCTTCCGCTGGCGGC 239

************************************************************

HpFMDref GCAACGACGAATAATAGTCCCTGGAGGTGACGGAATATATATGTGTGGAGGGTAAATCTG 300

HpFMD* GCAACGACGAATAATAGTCCCTGGAGGTGACGGAATATATATGTGTGGAGGGTAAATCTG 299

************************************************************

HpFMDref ACAGGGTGTAGCAAAGGTAATATTTTCCTAAAACATGCAATCGGCTGCCCCGCGACGGGA 360

HpFMD* ACAGGGTGTAGCAAAGGTAATATTTTCCTAAAACATGCAATCGGCTGCCCCGCAACGGGA 359

***************************************************** ******

HpFMDref AAAAGAATGACTTTGGCACTCTTCACCAGAGTGGGGTGTCCCGCTCGTGTGTGCAAATAG 420

HpFMD* AAAAGAATGACTTTGGCACTCTTCACCAGAGTGGGGTGTCCCGCTCGTGTGTGCAAATAG 419

************************************************************

HpFMDref GCTCCCACTGGTCACCCCGGATTTTGCAGAAAAATAGCAAGTTCCGGGGTGTCTCACTGG 480

HpFMD* GCTCCCACTGGTCACCCCGGATTTTGCAGAAAAACAGCAAGTTCCGGGGTGTCTCACTGG 479

********************************** *************************

HpFMDref TGTCCGCCAATAAGAGGAGCCGGCAGGCACGGAGTCTACATCAAGCTGTCTCCGATACAC 540

HpFMD* TGTCCGCCAATAAGAGGAGCCGGCAGGCACGGAGTCTACATCAAGCTGTCTCCGATACAC 539

************************************************************

HpFMDref TCGACTACCATCCGGGTCTCTCAGAGGGGGGAATGGCACTATAAATACCGCCTCCTTGCG 600

HpFMD* TCGACTACCATCCGGGTCTCTCAGAGAGGGGAATGGCACTATAAATACCGCCTCCTTGCG 599

************************** *********************************

HpFMDref CTCTCTGCCTTCATCAATCAAATC 624

HpFMD* CTCTCTGCCTTCATCAATCAAATC 623

************************

### HpMOX

HpMOXref CGACGCGGAGAACGATCTCCTCGAGCTGCTCGCGGATCAGCTTGTGGCCCGGTAATGGAA 60

HpMOX* CGACGCGGAGAACGATCTCCTCGAGCTGCTCGCGGATCAGCTTGTGGCCCGGTAATGGAA 60

************************************************************

HpMOXref CCAGGCCGACGGCACGCTCCTTGCGGACCACGGTGGCTGGCGAGCCCAGTTTGTGAACGA 120

HpMOX* CCAGGCCGACGGCACGCTCCTTGCGGACCACGGTGGCTGGCGAGCCCAGTTTGTGAACGA 120

************************************************************

HpMOXref GGTCGTTTAGAACGTCCTGCGCAAAGTCCAGTGTCAGATGAATGTCCTCCTCGGACCAAT 180

HpMOX* GGTCGTTTAGAACGTCCTGCGCAAAGTCCAGTGTCAGATGAATGTCCTCCTCGGACCAAT 180

************************************************************

HpMOXref TCAGCATGTTCTCGAGCAGCCATCTGTCTTTGGAGTAGAAGCGTAATCTCTGCTCCTCGT 240

HpMOX* TCAGCATGTTCTCGAGCAGCCATCTGTCTTTGGAGTAGAAGCGTAATCTCTGCTCCTCGT 240

************************************************************

HpMOXref TACTGTACCGGAAGAGGTAGTTTGCCTCGCCGCCCATAATGAACAGGTTCTCTTTCTGGT 300

HpMOX* TACTGTACCGGAAGAGGTAGTTTGCCTCGCCGCCCATAATGAACAGGTTCTCTTTCTGGT 300

************************************************************

HpMOXref GGCCTGTGAGCAGCGGGGACGTCTGGACGGCGTCGATGAGGCCCTTGAGGCGCTCGTAGT 360

HpMOX* GGCCTGTGAGCAGCGGGGACGTCTGGACGGCGTCGATGAGGCCCTTGAGGCGCTCGTAGT 360

************************************************************

HpMOXref ACTTGTTCGCGTCGCTGTAGCCGGCCGCGGTGACGATACCCACATAGAGGTCCTTGGCCA 420

HpMOX* ACTTGTTCGCGTCGCTGTAGCCGGCCGCGGTGACGATACCCACATAGAGGTCCTTGGCCA 420

************************************************************

HpMOXref TTAGTTTGATGAGGTGGGGCAGGATGGGCGACTCGGCATCGAAATTTTTGCCGTCGTCGT 480

HpMOX* TTAGTTTGATGAGGTGGGGCAGGATGGGCGACTCGGCATCGAAATTTTTGCCGTCGTCGT 480

************************************************************

HpMOXref ACAGTGTGATGTCACCATCGAATGTAATGAGCTGCAGCTTGCGATCTCGGATGGTTTTGG 540

HpMOX* ACAGTGTGATGTCACCATCGAATGTAATGAGCTGCAGCTTGCGATCTCGGATGGTTTTGG 540

************************************************************

HpMOXref AATGGAAGAACCGCGACATCTCCAACAGCTGGGCCGTGTTGAGAATGAGCCGGACGTCGT 600

HpMOX* AATGGAAGAACCGCGACATCTCCAACAGCTGGGCCGTGTTGAGAATGAGCCGGACGTCGT 600

************************************************************

HpMOXref TGAACGAGGGGGCCACAAGCCGGCGTTTGCTGATGGCGCGGCGCTCGTCCTCGATGTAGA 660

HpMOX* TGAACGAGGGGGCCACAAGCCGGCGTTTGCTGATGGCGCGGCGCTCGTCCTCGATGTAGA 660

************************************************************

HpMOXref AGGCCTTTTCCAGAGGCAGTCTCGTGAAGAAGCTGCCAACGCTCGGAACCAGCTGCACGA 720

HpMOX* AGGCCTTTTCCAGAGGCAGTCTCGTGAAGAAGCTGCCAACGCTCGGAACCAGCTGCACGA 720

************************************************************

HpMOXref GCCGAGACAATTCGGGGGTGCCGGCTTTGGTCATTTCAATGTTGTCGTCGATGAGGAGTT 780

HpMOX* GCCGAGACAATTCGGGGGTGCCGGCTTTGGTCATTTCAATGTTGTCGTCGATGAGGAGTT 780

************************************************************

HpMOXref CGAGGTCGTGGAAGATTTCCGCGTAGCGGCGTTTTGCCTCAGAGTTTACCATGAGGTCGT 840

HpMOX* CGAGGTCGTGGAAGATTTCCGCGTAGCGGCGTTTTGCCTCAGAGTTTACCATGAGGTCGT 840

************************************************************

HpMOXref CCACTGCAGAGATGCCGTTGCTCTTCACCGCGTACAGGACGAACGGCGTGGCCAGCAGGC 900

HpMOX* CCACTGCAGAGATGCCGTTGCTCTTCACCGCGTACAGGACGAACGGCGTGGCCAGCAGGC 900

************************************************************

HpMOXref CCTTGATCCATTCTATGAGGCCATCTCGACGGTGTTCCTTGAGTGCGTACTCCACTCTGT 960

HpMOX* CCTTGATCCATTCTATGAGGCCATCTCGACGGTGTTCCTTGAGTGCGTACTCCACTCTGT 960

************************************************************

HpMOXref AGCGACTGGACATCTCGAGACTGGGCTTGCTGTGCTGGATGCACCAATTAATTGTTGCCG 1020

HpMOX* AGCGACTGGACATCTCGAGACTGGGCTTGCTGTGCTGGATGCACCAATTAATTGTTGCCG 1020

************************************************************

HpMOXref CATGCATCCTTGCACCGCAAGTTTTTAAAACCCACTCGCTTTAGCCGTCGCGTAAAACTT 1080

HpMOX* CATGCATCCTTGCACCGCAAGTTTTTAAAACCCACTCGCTTTAGCCGTCGCGTAAAACTT 1080

************************************************************

HpMOXref GTGAATCTGGCAACTGAGGGGGTTCTGCAGCCGCAACCGAACTTTTCGCTTCGAGGACGC 1140

HpMOX* GTGAATCTGGCAACTGAGGGGGTTCTGCAGCCGCAACCGAACTTTTCGCTTCGAGGACGC 1140

************************************************************

HpMOXref AGCTGGATGGTGTCATGTGAGGCTCTGTTTGCTGGCGTAGCCTACAACGTGACCTTGCCT 1200

HpMOX* AGCTGGATGGTGTCATGTGAGGCTCTGTTTGCTGGCGTAGCCTACAACGTGACCTTGCCT 1200

************************************************************

HpMOXref AACCGGACGGCGCTACCCACTGCTGTCTGTGCCTGCTACCAGAAAATCACCAGAGCAGCA 1260

HpMOX* AACCGGACGGCGCTACCCACTGCTGTCTGTGCCTGCTACCAGAAAATCACCAGAGCAGCA 1260

************************************************************

HpMOXref GAGGGCCGATGTGGCAACTGGTGGGGTGTCGGACAGGCTGTTTCTCCACAGTGCAAATGC 1320

HpMOX* GAGGGCCGATGTGGCAACTGGTGGGGTGTCGGACAGGCTGTTTCTCCACAGTGCAAATGC 1320

************************************************************

HpMOXref GGGTGAACCGGCCAGAAAGTAAATTCTTATGCTACCGTGCAGCGACTCCGACATCCCCAG 1380

HpMOX* GGGTGAACCGGCCAGAAAGTAAATTCTTATGCTACCGTGCAGTGACTCCGACATCCCCAG 1380

****************************************** *****************

HpMOXref TTTTTGCCCTACTTGATCACAGATGGGGTCAGCGCTGCCGCTAAGTGTACCCAACCGTCC 1440

HpMOX* TTTTTGCCCTACTTGATCACAGATGGGGTCAGCGCTGCCGCTAAGTGTACCCAACCGTCC 1440

************************************************************

HpMOXref CCACACGGTCCATCTATAAATACTGCTGCCAGTGCACGGTGGTGACATCAATCTAAAGTA 1500

HpMOX* CCACACGGTCCATCTATAAATACTGCTGCCAGTGCACGGTGGTGACATCAATCTAAAGTA 1500

************************************************************

HpMOXref CAAAAACAAA 1510

HpMOX* CAAAAACAAA 1510

**********

### CbFLD1

CbFLD1ref GGATCCCTTCAACAGCGGAGTCTCAAACATTGGCTATTATCAGTGTATTTAATTACTGAT 60

CbFLD1* GGATCCCTTCAACAGCGGAGTCTCAAGCAGTGGCTATTATCAGTGTATTTAATTACTGAT 60

************************** ** ******************************

CbFLD1ref GCATTGTATTATAGTGCATACATAGTTAATGATTATTCTCTGTTATCACTGAAAATTTTG 120

CbFLD1* GCATTGTATTATAGTGCATACATAGTTAATAATTACTCTCTGTTATCATTGAAAATTTTG 120

****************************** **** ************ ***********

CbFLD1ref AAATTCTCACTCTCACGCAG--CAAAACTTTGCCTAATTGAGTAAGTGGAACGCAATATT 178

CbFLD1* AAATTCTCACTCTCACGCAGTGCAAAACTTTGCCTAATTGAGTAAGTGGAACGCAATATT 180

******************** **************************************

CbFLD1ref TAGGCTACATATTTTGGATTCCCTTAAGTATGTAATCAAAGATCATTCATACTGCCATCT 238

CbFLD1* TAGGCTACATATTTTGGATTCCCTTAAGTATGTAATCAAAGATCATTCATACTGCCATCT 240

************************************************************

CbFLD1ref TATAATATTGGAGTATTATTATGTTGCTATACTGTTCTACCTGTTTATTCTAT-GTATGC 297

CbFLD1* TATAATATTGGAGTATTATTATGTTGCTATACTGTTCTACCTGTTTATTCTATTGTATGC 300

***************************************************** ******

CbFLD1ref GTCTAAATCTTTCTATCAGTTTCTATACTATCTTTCGT--GCAATGAAATATTACTCCAA 355

CbFLD1* GTCTAAATCTTTCCATCAGTTTCTATACTATCTTTCGTTTGCAATGAAATATTACTCCAA 360

************* ************************ ********************

CbFLD1ref TTCGCTTGTTTCAACTCGCTTGCCTTCTCTCTTGCCTTCTTCTCTTTCTCTCTTTTTTTC 415

CbFLD1* TTCGCTTGTTTCAACTCGCTTGCCTTCTCTCTTGCCTTCTT-------------TTTTTC 407

***************************************** ******

CbFLD1ref TTTTCATTTTATCGTTGTTTAAACGGTCTATAAATATGTAACGTTGTCGCTTAGTTTTAA 475

CbFLD1* TTTTCATTTTATCGTTGTTTAAACGGTATATAAATATGTAACGTTGTCGCTTAGTTTTGA 467

*************************** ****************************** *

CbFLD1ref TAAATCACTTTTGTTGCTCTCAATTCTGTTTTGACATATTAAGGTTAGTCAATTGATTGA 535

CbFLD1* GAAATCACTTTTGTTGCTCTCAATTCTGTTTTGACATCTTAAGGTTAGTCAATTGATTGA 527

************************************ **********************

CbFLD1ref ATCAACTACACTAAATCATATTTATCTATTTTTTATTCCACAAAA 580

CbFLD1* ATCAACTACACTAAATCATATTTATCTATTTTTTATTCCACAAAA 572

*********************************************

### CbAOD1

CbAOD1ref GGAGTATACGTAAATATATAATTATATATAATCATATATATGAATACAATG-----AAAG 55

CbAOD1* GGAGTATACGTAAATATATAATTATATATAATCATATATATGAATACAATGCAATGAAAG 60

*************************************************** ****

CbAOD1ref TAAATATGATAAGATTGAAATAATAACAAACAGCGATAAATATATCTCAAAATGGAGTTA 115

CbAOD1* TGAATATGATAAGATTGAAATAATAACAAACAGCGATAAATATATCTCAAAATGGAGTTA 120

* **********************************************************

CbAOD1ref CACAACAAATAATAATAAAATATAAATTATAAAATATAAA-------GGAATAAAA--TA 166

CbAOD1* CACAACAAATAATAATAAAATATAAATTATAAATTATAAATTATAAAAGAATAAAAAATA 180

********************************* ****** ******** **

CbAOD1ref AACCCCACTAATTTATTTTATTAAAAGATAGATTGGTATCTTTACTTAATAACAATTCTG 226

CbAOD1* AACCCCACTAATTTATTTTATTAAAAGATAGATTGGTATCTTTACTTAATAACAATTCTG 240

************************************************************

CbAOD1ref AAACTTTATTCACTTAATTTTATTTAACTTATTTAATTTATTTTTACCCCAGTTTTTTCA 286

CbAOD1* AAACTTTATTCACTTAATTTTATTTAACTTATTTAATTTATTTTTACCCCAGTTTTT-CA 299

********************************************************* **

CbAOD1ref GTACAGTGCAGCTCCGAAACTTTATTTGGCTGTGATTTGGCTGTGATTTGGCT------- 339

CbAOD1* GTACAATGCAGCTCCGAAACTTTATTTGGCTGTGATTTGGCTGTGATTTGGCTGTGATTT 359

***** ***********************************************

CbAOD1ref ----TGGCTTGGCTGGCTGGAATTGTCTCCTGCAGGAATTGCTCGGGGTCCGGTTCTCCC 395

CbAOD1* GGCTTGGCTTGGCTGGCTGGAATTGTCTCCTGCAGGAATTGCTCGGGGTCCGGTTCTCCC 419

********************************************************

CbAOD1ref GC-AGCTGGATATTTGGCTGGCTG-CT--------------CTGTCTGGCTGCTCTGCCA 439

CbAOD1* GCTGGCTGGCTATTTGGCGGGCTGGCTATTTGGCGGGCTGGCTGGCTGGCTGCTCTGCCA 479

** ***** ******** ***** ** *** ***************

CbAOD1ref TCTGCTGTGGCCACCCCCGCATCTCTGGATGCACGCCGTGCAGCTGGACTTGCGTCTACC 499

CbAOD1* TCTGCTGTGGCCACCCC-GCATCTCTGGATGCACGCCGTGCAGCTGGACGTGCGTCTACC 538

***************** ******************************* **********

CbAOD1ref CTGCAGCCGTGTGCCTCATCTCCCAATCTCTCAATCAGCCAGTCAGCCAGCCAGCCAAAA 559

CbAOD1* CTGCAGCCGTGTGCCTTATTTCCCAATCTCCCAATCTCTCAATCTGCCAGTCAGCCAAAA 598

**************** ** ********** ***** ** ** ***** *********

CbAOD1ref TACGGGCCAGGCAGGCAGGCAGGCAGGCAGGCAGGCAGGCAGGCAGGCAGGCAGGCAGTG 619

CbAOD1* CACCGGCCAGGCAGGCAGGCAGGCAGGCAGGCAGGCAG--------------------TG 638

** ********************************** **

CbAOD1ref ATGCCTTCCCACGCCCCACCCCGCATAAACATCCCCAGCAGTTTCCCCAGCAGTTTCCCC 679

CbAOD1* AAGCCTTCCCACGCCCCACTCCGCATAAACATCCCCAGCAGTTTCCCCAGCAGTTTCCCC 698

* ***************** ****************************************

CbAOD1ref AGCTTTTCAATTTAATAAAATAGCCTGTTTCTGTTTCTGTTTTATATTATACAATTTTTT 739

CbAOD1* AGCTTTTCAATTTAATAAAATAGCCTGTTTCTGTTTCTGTTTTATATTATACAATTTTTT 758

************************************************************

CbAOD1ref ATCCTAATAATTACTCTTTTGGGAATTAAATAATAATTATATCATATACCCATATCACAT 799

CbAOD1* ATCCTAATAATTACTCTTTCGGGAATTAAATAATAATTATATCATATACCCATATCACAT 818

******************* ****************************************

CbAOD1ref TTTACTATATTTACTATCTATAAATAATTTCATATTATAATATTAATTTATATTCGCTTA 859

CbAOD1* TTTACTATATTTACTATCTATAAATAAATTCATATTATAATATTAATTTATATTCGCTTA 878

*************************** ********************************

CbAOD1ref ATTAAAATGCTCTTTTCCATCATCATCATCATCATCATCA---CGAGTTTTCGGTTATCA 916

CbAOD1* ATTAAAATGCTCTTTTCCATCATCATCATCATCATCATCATCACGAGTTTTCGGTTATCA 938

**************************************** *****************

CbAOD1ref ATACTCTTTTCATTAACTTCTAGAATTTCATTATTTATTTTTTATTGACTGGAAATTTTC 976

CbAOD1* ATACTCTTTTCATTAATTTCTAGAATTTCATTATTTATTTTTTATTGACTGGAAATTTTC 998

**************** *******************************************

CbAOD1ref AATCAATTTTATTTATTTTTATTTATTTATTTTCATATTCTTAGATTTAAACTTTTTAGA 1036

CbAOD1* AATCAATTTTATTTATTTTTATTTATTTATTTTCATATTCTTAGATTTAAACTTTTTAGA 1058

************************************************************

CbAOD1ref TGACCGCTATTTTACTTACTTACTTACTTACTTACTTACTTACTTACTTACATACCTACT 1096

CbAOD1* TGACCGCTATTTTACTTACTTACTTACT-------------------------------- 1086

****************************

CbAOD1ref TACTGTGATTTTATAATATGATAAGAATTAATTTTCATATTTATGATGATG------TAA 1150

CbAOD1* ----GTTGTTTTATATTATGATAAGAATTAATTTTCATATTTATGATGATGATGATGTAA 1142

** ******* *********************************** ***

CbAOD1ref ATTTAACCTAGTATACTATTTTAAAGTTATCACTATCTTTTAGTGCTGGCATTTTTTATT 1210

CbAOD1* ATTTAACCTAGTATACTATTTTAAAGTTATCACTATCTTTTAGTGCTGGCATTTTTTATT 1202

************************************************************

CbAOD1ref CTATTTTCATATATGTATATAAGTAAATTAAGTATCATCACGCTGCTTACTGTACGTTTA 1270

CbAOD1* CTATTTTCATATATGTATATACGTAAATTAAGTATCATCACGCTGCTTACTGTACGTTTA 1262

********************* **************************************

CbAOD1ref AAATGTGGAGATGGAAATAGAGATGGGGATGAAGATGAAGATGATGAGAATTATAAACCA 1330

CbAOD1* AAATGTGGAGATGGAAATAGAGATGGGGATGAAGATGAAGATGATGAGAATTATAAACCA 1322

************************************************************

CbAOD1ref TTCATTCATTAATCAATCAATATAACTTATAAAAAAATTTATATTTAAATGAATTAATTT 1390

CbAOD1* TTCATTCATTAATCAATCAATATAACTTATAAAAAAATTTATATTTAAATGAATTAATTT 1382

************************************************************

CbAOD1ref CCTTTATTTTAATAATATCGTTAATTCTTTTAAATTCTATTTTATTTTAATTCTTTCTTT 1450

CbAOD1* CCTTTATTTTAATAATATCGTTAATTCTTTTAAATTCTATTTTATTTTAATTCTTTCTTT 1442

************************************************************

CbAOD1ref ATCATAGTTATCATATAACAATTATATAACATAGATACACAATTATTATTTTATTATCAT 1510

CbAOD1* ATCATAGTTATCATATAACAATTATATAACATAGATACACAATTATTATTTCATTATCAT 1502

*************************************************** ********

CbAOD1ref ATTATTTTTTAAAATATTGATTATTTTTAAAATAATATCTTAATTAATTAATTTTTACGA 1570

CbAOD1* ATTATTTTTTAAAATATTGATTATTTTTAAAATAATATCTTAATTAATTAATTTTTACGA 1562

************************************************************

CbAOD1ref ATATACAAATTTTAACGACTTTCTTTTTTTAACGAATTTTAACGAACTTTTAAAAAAACA 1630

CbAOD1* ATATACAAATTTTAACGACTTACTTTTTTTAACGAATTTTAACGAACTTTTAAAAAAACA 1622

********************* **************************************

CbAOD1ref AAAAAAAAAAAACAAAATTATTTTTCAATA 1660

CbAOD1* AAAAAAAAAAAACAAAATTATTTTTCAATA 1652

******************************

### PmMOD1

PmMOD1ref CGAGATGGTACATACTTAAAAGCTGCCATATTGAGGAACTTCAAAGTTTTATCTGTTTTT 60

PmMOD1* CGAGATGGTACATACTTAAAAGCTGCCATATTGAGGAACTTCAAAGTTTTATCTGTTTTT 60

************************************************************

PmMOD1ref AGAATTAAAAGACGATTGTTGTAACAAAACGTTGTGCCTACATAAACTCAAATTAATGGA 120

PmMOD1* AGAATTAAAAGACGATTGTTGTAACAAAACGTTGTGCCTACATAAACTCAAATTAATGGA 120

************************************************************

PmMOD1ref AATAGCCTGTTTTGAAAAATACACCTTCTTAAGTACTGACAAAGTTTTGTTAAATGACTA 180

PmMOD1* AATAGCCTGTTTTGAAAAATACACCTTCTTAAGTACTGACAAAGTTTTGTTAAATGACTA 180

************************************************************

PmMOD1ref TCGAACAAGCCATGAAATAGCACATTTCTGCCAGTCACTTTTAACACTTTCCTGCTTGCT 240

PmMOD1* TCGAACAAGCCATGAAATAGCACATTTCTGCCAGTCACTTTTAACACTTTCCTGCTTGCT 240

************************************************************

PmMOD1ref GGTTGACTCTCCTCATACAAACACCCAAAAGGGAAACTTTCAGTGTGGGGACACTTGACA 300

PmMOD1* GGTTGACTCTCCTCATACAAACACCCAAAAGGGAAACTTTCAGTGTGGGGACACTTGACA 300

************************************************************

PmMOD1ref TCTCACATGCACCCCAGATTAATTTCCCCAGACGATGCGGAGACAAGACAAAACAACCCT 360

PmMOD1* TCTCACATGCACCCCAGATTAATTTCCCCAGACGATGCGGAGACAAGACAAAACAACCCT 360

************************************************************

PmMOD1ref TTGTCCTGCTCTTTTCTTTCTCACACCGCGTGGGTGTGTGCGCAGGCAGGCAGGCAGGCA 420

PmMOD1* TTGTCCTGCTCTTTTCTTTCTCACACCGCGTGGGTGTGTGCGCAGGCAGGCAGGCAGGCA 420

************************************************************

PmMOD1ref GCGGGCTGCCTGCCATCTCTAATCGCTGCTCCTCCCCCCTGGCTTCAAATAACAGCCTGC 480

PmMOD1* GCGGGCTGCCTGCCATCTCTAATCGCTGCTCCTCCCCCCTGGCTTCAAATAACAGCCTGC 480

************************************************************

PmMOD1ref TGCTATCTGTGACCAGATTGGGACACCCCCCTCCCCTCCGAATGATCCATCACCTTTTGT 540

PmMOD1* TGCTATCTGTGACCAGATTGGGACACCCCCCTCCCCTCCGAATGATCCATCACCTTTTGT 540

************************************************************

PmMOD1ref CGTACTCCGACAATGATCCTTCCCTGTCATCTTCTGGCAATCAGCTCCTTCAATAATTAA 600

PmMOD1* CGTACTCCGACAATGATCCTTCCCTGTCATCTTCTGGCAATCAGCTCCTTCAATAATTAA 600

************************************************************

PmMOD1ref ATCAAATAAGCATAAATAGTAAAATCGCATACAAACGTCATGAAAAGTTTTATCTCTATG 660

PmMOD1* ATCAAATAAGCATAAATAGTAAAATCGCATACAAACGTCATGAAAAGTTTTATCTCTATG 660

************************************************************

PmMOD1ref GCCAACGGATAGTCTATCTGCTTAATTCCATCCACTTTGGGAACCGTTCTCTCTTTACCC 720

PmMOD1* GCCAACGGATAGTCTATCTGCTTAATTCCATCCACTTTGGGAACCGTTCTCTCTTTACCC 720

************************************************************

PmMOD1ref CAGATTCTCAAAGCTAATATCTGCCCCTTGTCTATTGTCCTTTCTCCGTGTACAAGCGGA 780

PmMOD1* CAGATTCTCAAAGCTAATATCTGCCCCTTGTCTATTGTCCTTTCTCCGTGTACAAGCGGA 780

************************************************************

PmMOD1ref GCTTTTGCCTCCCATCCTCTTGCTTTGTTTCGGTTATTTTTTTTTTCTTTTGAAACTCTT 840

PmMOD1* GCTTTTGCCTCCCATCCTCTTGCTTTGTTTCGGTTATTTTTTTTT-CTTTTGAAACTCTT 839

********************************************* **************

PmMOD1ref GGTCAAATCAAATCAAACAAAACCAAACCTTCTATTCCATCAGATCAACCTTGTTCAACA 900

PmMOD1* GGTCAAATCAAATCAAACAAAACCAAACCTTCTATTCCATCAGATCAACCTTGTTCAACA 899

************************************************************

PmMOD1ref TTCTATAAATCGATATAAATATAACCTTATCCCTCCCTTGTTTTTTACCAATTAATCAAT 960

PmMOD1* TTCTATAAATCGATATAAATATAACCTTATCCCTCCCTTGTTTTTTACCAATTAATCAAT 959

************************************************************

PmMOD1ref CTTCAAATTTCAAATATTTTCTACTTGCTTTATTACTCAGTATTAACATTTGTTTAAACC 1020

PmMOD1* CTTCAAATTTCAAATATTTTCTACTTGCTTTATTACTCAGTATTAACATTTGTTTAAACC 1019

************************************************************

PmMOD1ref AACTATAACTTTTAACTGGCTTTAGAAGTTTTATTTAACATCAGTTTCAATTTACATCTT 1080

PmMOD1* AACTATAACTTTTAACTGGCTTTAGAAGTTTTATTTAACATCAGTTTCAATTTACATCTT 1079

************************************************************

PmMOD1ref TATTTATTAACGAAATCTTTACGAATTAACTCAATCAAAACTTTTACGAAAAAAAAATCT 1140

PmMOD1* TATTTATTAACGAAATCTTTACGAATTAACTCAATCAAAACTTTTACGAAAAAAAAATCT 1139

************************************************************

PmMOD1ref TACTATTAATTTCTCAAA 1158

PmMOD1* TACTATTAATTTCTCAAA 1157

******************

### PmMOD2

PmMOD2ref GGATCCACTACAGTTTACCAATTGATTACGCCAATAGTGTTTATTTCACCAAGTAATTAC 60

PmMOD2* GGATCCACTACAGTTTACCAATTGATTACGCCAAT-GTGTTTATTTCACCAAGTAATTAC 59

*********************************** ************************

PmMOD2ref AAAACTGAGATTTGGTTATGTCATTATGTATTTTCGGCAATGGCTGTAATTTAAACTGGA 120

PmMOD2* AAAACTGAGATTTGGTTATGTCATTATGTATTTTCGGCAATGGCTGTAATTTAAACTGGA 119

************************************************************

PmMOD2ref TTAGGGTTAATTAACGTTTAGCCTACGAAAGCGGCTAGCTTTTATTTCTGCTTTTGTTTT 180

PmMOD2* TTAGGGTTAATTAACGTTTAGCCTACGAAAGCGGCTAGCTTTTATTTCTGCTTTTGTTTT 179

************************************************************

PmMOD2ref GAGCCCGTTTCTAATTCCACATCTTTGCAATTTCGTTCCATCTTTTAAAATTAAGTGCTC 240

PmMOD2* GAGCCCGTTTCTAATTCCA-ATCTTTGCAATTTCGTTCCATCTTTTAAAATTAAGTGCTC 238

******************* ****************************************

PmMOD2ref TTTTCTAATCTGATCAAAGATCAAGCCATCGTAGAGTCAAGTAAAACAAAATAATGTACT 300

PmMOD2* TTTTCTAATCTGAT-AAAGAT-AAGCCATCGTAGAGT-AAGTAAAACAAAATAATGTACT 295

************** ****** *************** **********************

PmMOD2ref GTATATTAAGCGGAAAAACTTGGAAGAAGTCGTATGATGTTGAAGGAGCAAAGAATCGAC 360

PmMOD2* GTATATTAAGCGGAAAAACTTGGAA-AAGTCGTATGATGTTGAAGGAGCAAAGAAT-GAC 353

************************* ****************************** ***

PmMOD2ref TAATATTAGGAGATTTAAGCAAACAATGTTGAGGGGAACAGGACGATTAACCCCTTATAG 420

PmMOD2* TAATATTAGGAGATTTAAGCAAACAATGTTGAGGGGAACAGGACGATTAACCCCTTATAG 413

************************************************************

PmMOD2ref AGGAAGCGTCTTCTGATGTGCGAAGGGGGAGGGGTCAAAAGCACTGAGCAGTGCTAATTA 480

PmMOD2* AGGAAGCGTCTT-TGATGTTCGAAGGGGGAGGGGTCAAAAGCACTGAGCAGTGCTAATTA 472

************ ****** ****************************************

PmMOD2ref GTAACCAATTTCTGTAAGCAATGAAACTTGTTGCTATTGGAAATACTATTAAGTAATACA 540

PmMOD2* GTAACCAATTTCTGTAAGCAATGAAACTTGTTGCTATTGGAAATACTATTAAGTAATACA 532

************************************************************

PmMOD2ref AGGTACAGACTAATGGGGGTGAGCCGGTAGTTCAGGCTATCTTATCAGACAGACTATGTC 600

PmMOD2* AGGTACAGACTAATGGGGGTGAGCCGGTAGTTCAGGCTATCTTAT-AGACAGACTAT-TC 590

********************************************* *********** **

PmMOD2ref CGGAGTGTCTAATCATTTGGTGCACCTGGTTGGATAGTTATCAGTCAACTGCTTTACGGT 660

PmMOD2* CGGATTGTCTAATCATT-GGTGCACCTGGTTA-ATAATTATCAGTCAACTCTTTTACGGT 648

**** ************ ************* *** ************* ********

PmMOD2ref GCTGATAGGTCTT-GCGAACTTGCGCTTGTGGAATTTGGTTGTTAATCAAACTGTTCTGT 719

PmMOD2* GCTGATAGGTCTTTGCGAACTTGCCCTTGTGGAATTTGGTTGTTAATCAAACTGTTCTGT 708

************* ********** ***********************************

PmMOD2ref ATTGCATGTCATACTACTATTGATATAGTTAATGTGGACTTACTCATCTGGCCATTTGCA 779

PmMOD2* ATTTCATGTCATACTACTATTGATATTATTAATGTT-ACTTACTCATCTGGCCATTT--A 765

*** ********************** ******* ******************** *

PmMOD2ref ACAGGTTTGAAGCTTTAATGCTCTTAACTAACAGCAATCCATCACCGTCAACCTTAACCC 839

PmMOD2* ACAGGTTTGAAGCTTTAATGCTCTTAACTAACAGCAATCCATCACCGTCAACCTTAACCC 825

************************************************************

PmMOD2ref CCCTGGTGCTTGCTGTCTTTATCCTTCGTATCTTTTTCATGTTGCACCGCCCTGTTCCTT 899

PmMOD2* CCCTGGTGCTTGCTGTCTTTATCCTTCGTATCTTTTTCATGTTGCACCGCCCTGTTCCTT 885

************************************************************

PmMOD2ref ATACGGTTGTTCCCCCATAGGCTAACTTCTCTGTTTCCGACCATCTCTGGCAATAACAAA 959

PmMOD2* ATACGGTTGTTCCCCCATAGGCTAACTTCTCTGTTTCCGACCATCTCTG-CAATAACAAA 944

************************************************* **********

PmMOD2ref GAATTCTATAGCGCTTACACTATAATCATACAATGACTCTACATGCCATTTTCACTTTAC 1019

PmMOD2* GAATTCTATA-CGCTTACACTATAATCATACAATGACTCTACATGCCATTTTCACTTTAC 1003

********** *************************************************

PmMOD2ref TTACTCGCCATCGGAAGATACTGAATCAGAAAGCCATAGTAATCTACATAACTTCAAAAC 1079

PmMOD2* TTACTTGCCATCGGAAGATACTGAATCAGAAAGCCATAGTAA-CTACATAACTTCAAAAC 1062

***** ************************************ *****************

PmMOD2ref ACACCCTTTTTACAGTATTAGTTACAATTTTGCCAATGTGTGTTTGATAACCCAAGGTGG 1139

PmMOD2* ACACCCTTTTTACAG-ATTAGTTACAATTTTGTCAATGTTTGTTTGATAACCCAAGGTGG 1121

*************** **************** ****** ********************

PmMOD2ref AACGTTTCCAGTTAGACCTGTTCAATCCAACTCACTTTACCACCCCAAAACTTTCCTACC 1199

PmMOD2* AACGTTTCCAGTTAGACCTGTTTAATCCAACTCACTTTACCACCCCAAAACTTTCCTACC 1181

********************** *************************************

PmMOD2ref GTTAGACAAATACTGGCTAAATCTGACGAAAACAACCAATCAACAATTGAATCCACTGGG 1259

PmMOD2* GTTAGACAAATACTGGCTAAATCTGACGAAAACAACCAATCAACAATTGAATCCACTGGG 1241

************************************************************

PmMOD2ref AGGTATCTCTAATCCACTGACAAACTTTGCTAAAACAAGAAAAAGTGGGGGCCTCCGTTG 1319

PmMOD2* AGGTATCTCTAATCCACTGACAAACTTTGCTAAAACAAGAAAAAGTGGGGGCCTCCGTTG 1301

************************************************************

PmMOD2ref CGGAGAAGACGTGCGCAGGCTTAAAAACACAAGACGAACACTTGGAAGTACCCCAGATTT 1379

PmMOD2* CGGAGAAGACGTGCGCAGGCTTAAAAACACAAGA-GAACACTTGGAAGTACCCCAGATTT 1360

********************************** *************************

PmMOD2ref TTAGCTTCCTACTATACTGACACCCCCTATTCAAGCACGACGGTGATTGATTCATTCAAT 1439

PmMOD2* TTAGCTTCCTACTATTCTGACACCCCCTATTCAAGCACGACGGTGATTGATTCATTCAAT 1420

*************** ********************************************

PmMOD2ref TTTGCTGCTCCAATGATAGGATAAACCCTTTTAAACTTCAATCAGACCTCTGTCCTCCAT 1499

PmMOD2* TTTGCTGCTCCAATGATAGGATAAACCCTTTTGGACTTCAATCAGACCTCTGTCCTCCAT 1480

******************************** **************************

PmMOD2ref AGCAATATAAAAACCTTCTAGGTTGCCCCACTTCCTCTCTCCTGTACTGCCCCAATGAGT 1559

PmMOD2* AGCAATATAAATACCTTCTAG-TTGCCCCACTTCCTCTCTCCTGTACTGCCCCAATGAGT 1539

*********** ********* **************************************

PmMOD2ref GACTTATTCAAGTTACTTTCTCTCTTTTCCTAACAATTAAACAAGAAGCTTTATTATAAC 1619

PmMOD2* GACTTATTCAAGTTACTTTCTCTCTTTTCCTAACAATTAAACAAGAAGCTTTATTATAAC 1599

************************************************************

PmMOD2ref ATTAATATACTATTTTATAACAGGATTGAAAATTATATTTATCTATCTAAAACTAAAATT 1679

PmMOD2* ATTAATATACTATTTTATAACAGGATTGAAA-TTATATTTATCTATCTAAAACTAAAATT 1658

******************************* ****************************

PmMOD2ref CAAA 1683

CbAOD1* CAAA 1662

****

## S 3 – Screening and rescreening data

S 3: Screening and rescreening data of the transformants to obtain the reporter strains generated in this study. The underlying strategy is described in the materials and methods section. In short, to avoid clonal variation biasing the promoter strength assessments (e.g. by copy number variation, different integration sites or genomic alterations (Vogl et al. 2018a)), we screened for each promoter construct 42 transformants followed by a rescreening of 4 transformants (in replicates). The 4 transformants used for the rescreening are highlighted with red rectangles. Additionally, the untransformed wildtype (Wt) strain is included as reference for background fluorescence. Note the different scaling of the y-axis, as the promoters showed variable strengths. As the relative expression performance of the same strain was identical between derepressed and methanol induced conditions, in part data from representative condition is shown.

### GFP screening

#### HpFMD promoter

##### Derepressed conditions


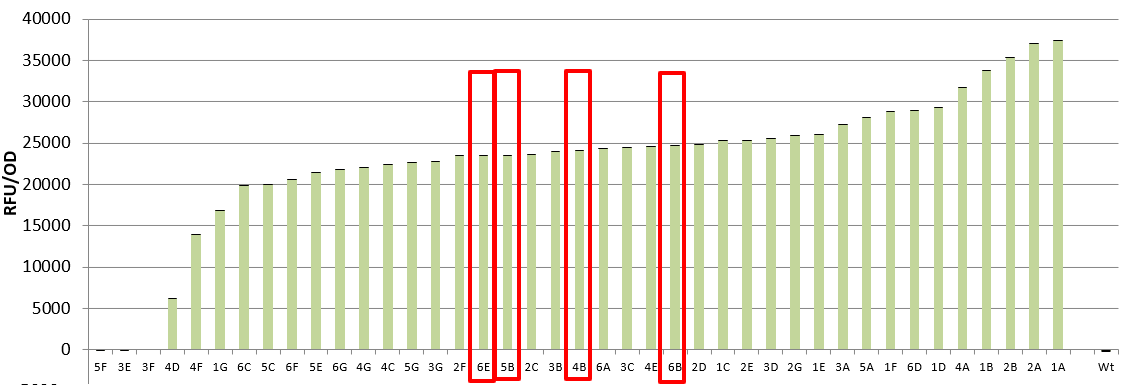


##### Methanol induction


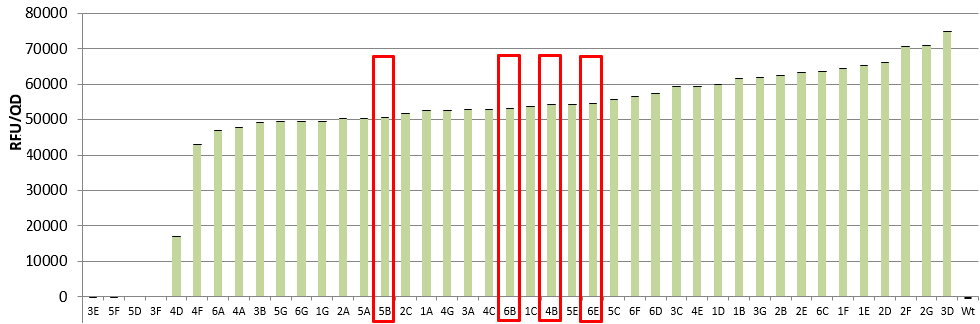


#### HpMOX promoter

##### Derepressed conditions


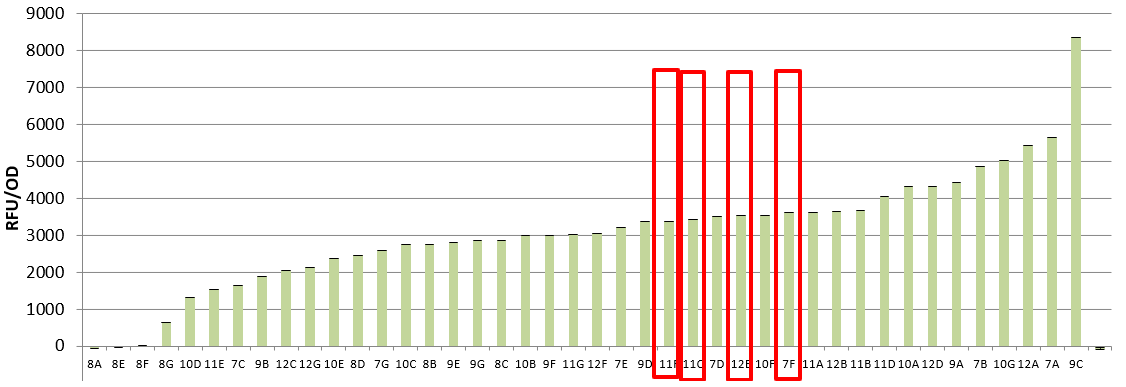


##### Methanol induction


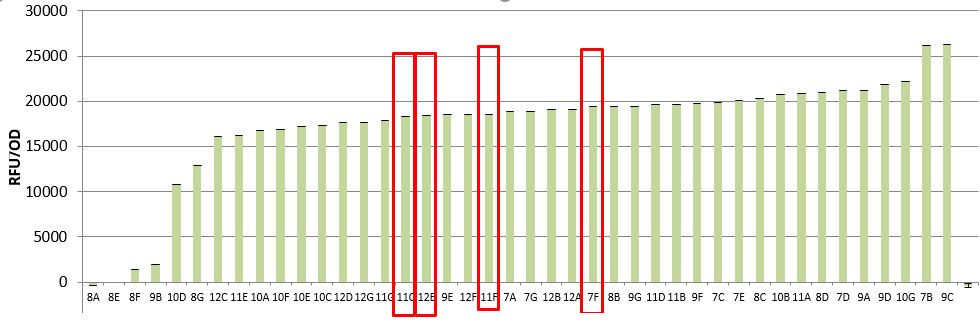


#### CbFLD1 promoter

##### Derepressed conditions


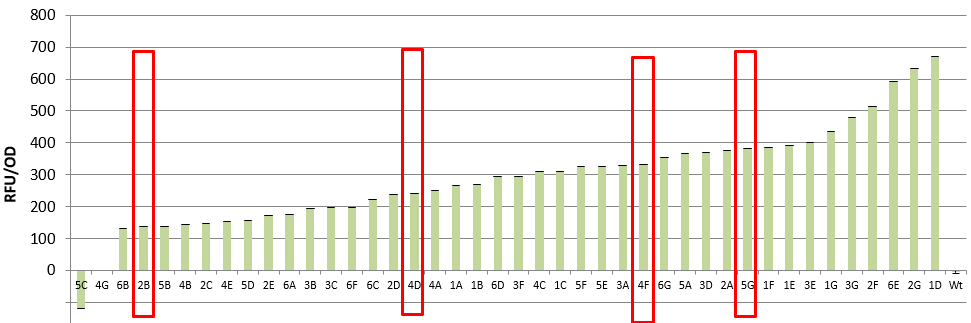


##### Methanol induction


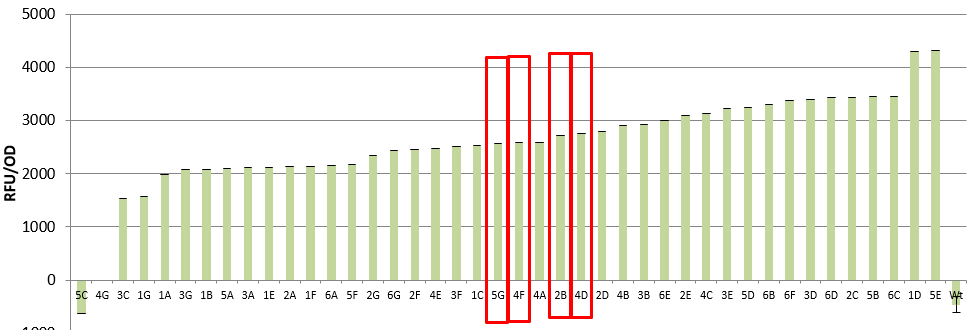


#### CbAOD1 promoter

##### Derepressed conditions


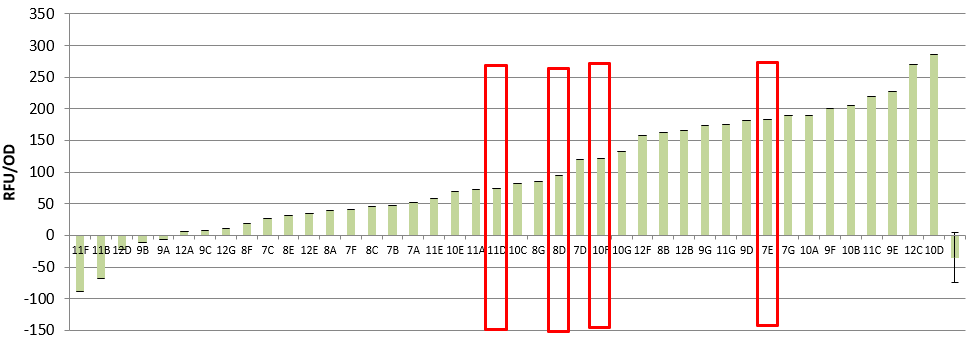


##### Methanol induction


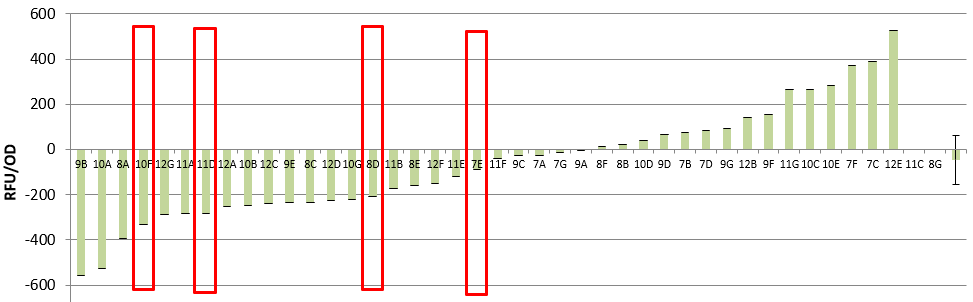


#### PmMOD1

##### Derepressed conditions


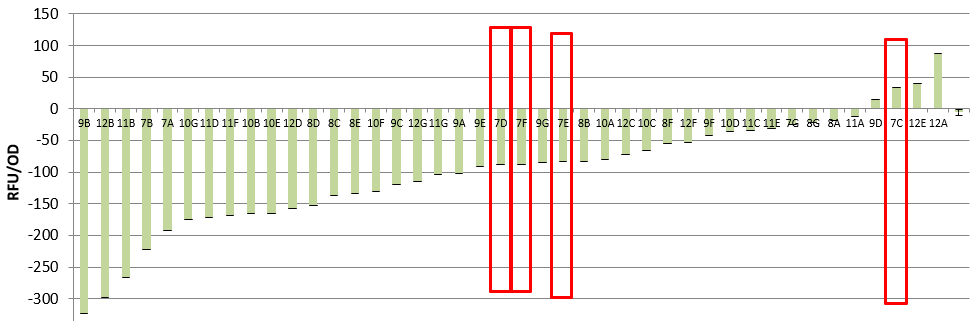


##### Methanol induction


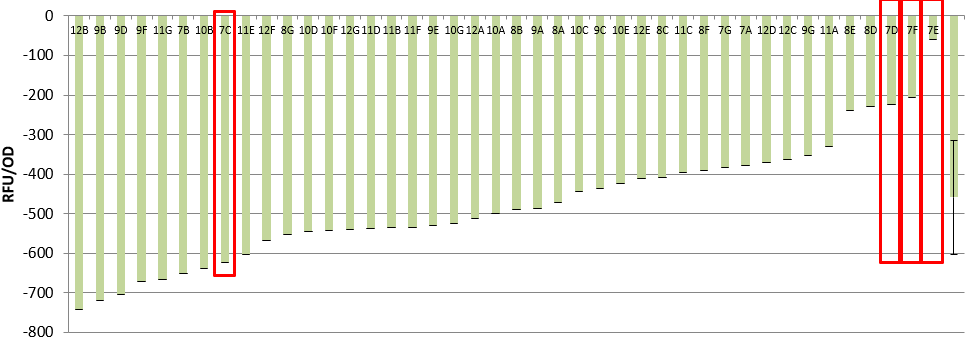


#### PmMOD2

##### Derepressed conditions


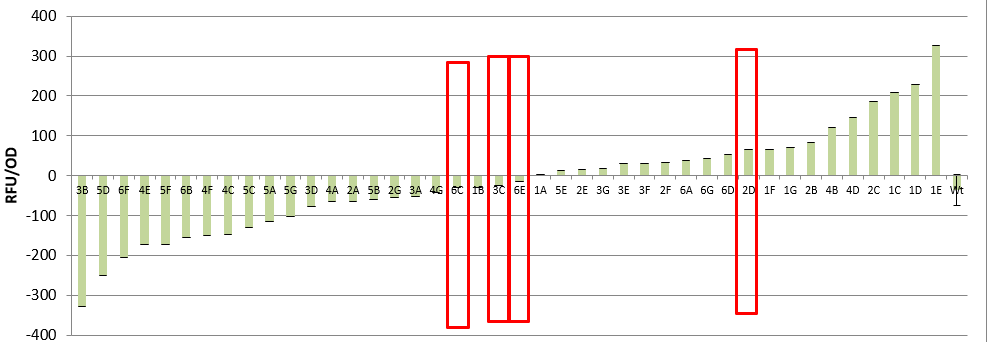


##### Methanol induction


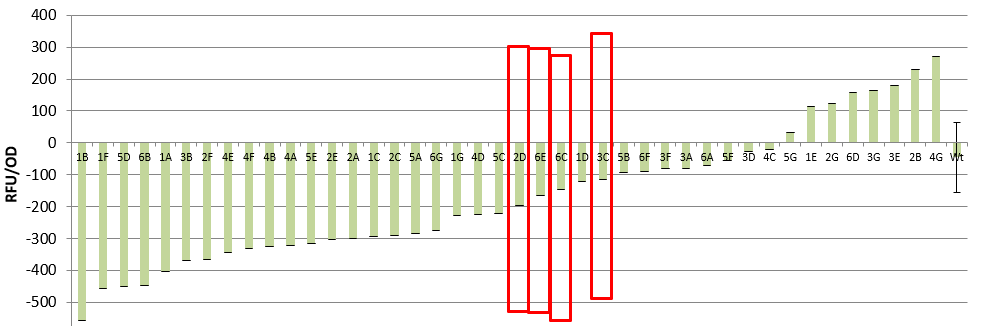


### GFP rescreening

#### HpFMD, HpMOX, CbFLD1

##### Derepressed conditions


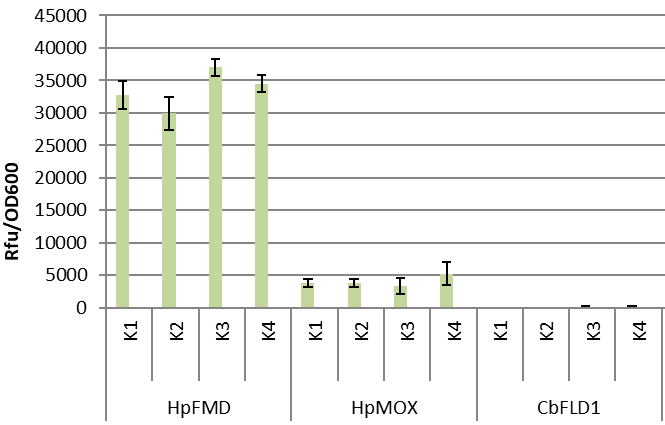


##### Methanol induction


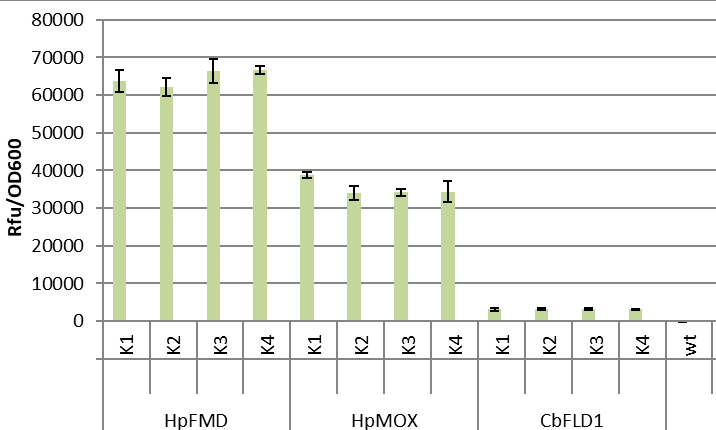


#### PmMOD1, PmMOD2

##### Derepressed conditions


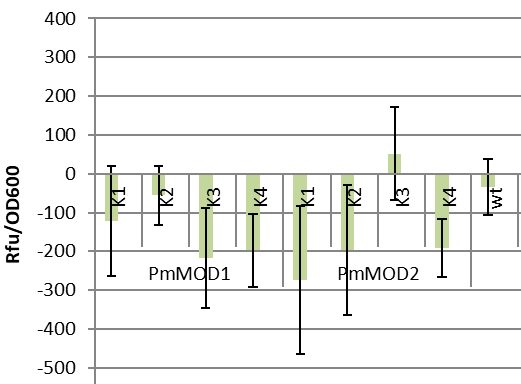


##### Methanol induction


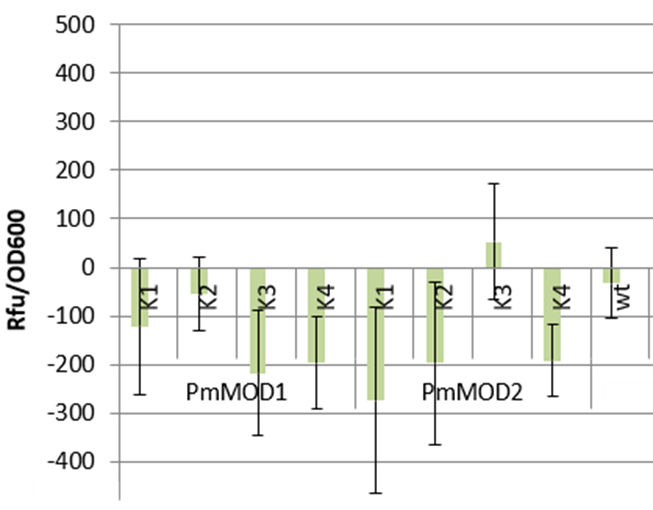


#### CbAOD1

##### Derepressed conditions


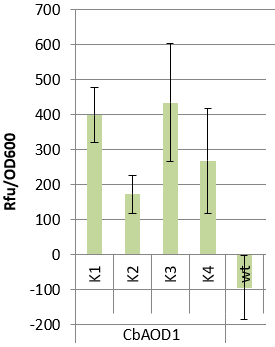


### HRP

#### Screening


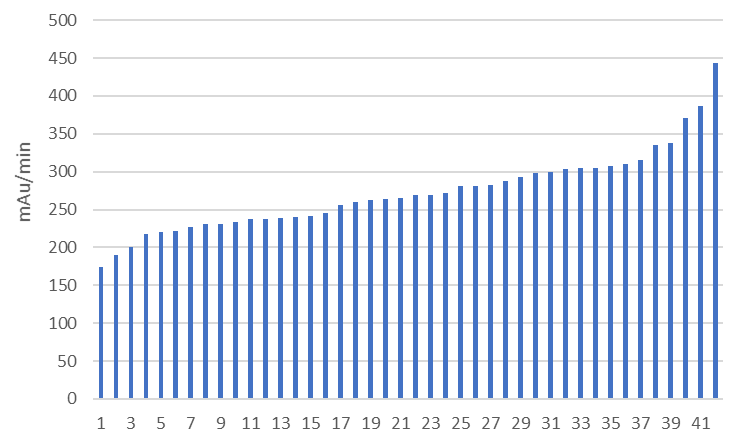


Rescreening


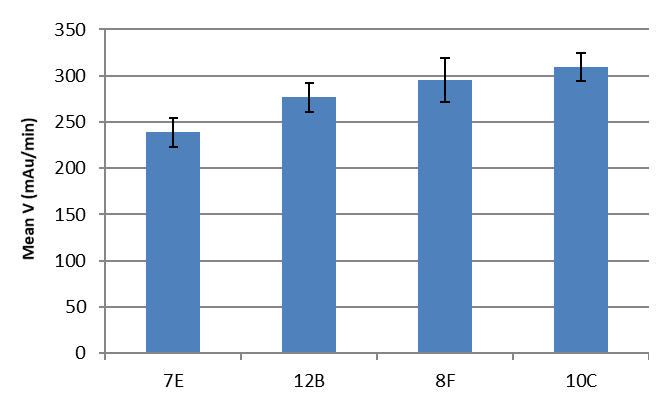


### CalB

#### Screening


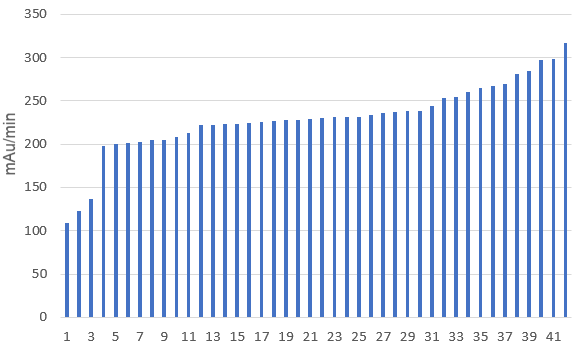


Rescreening


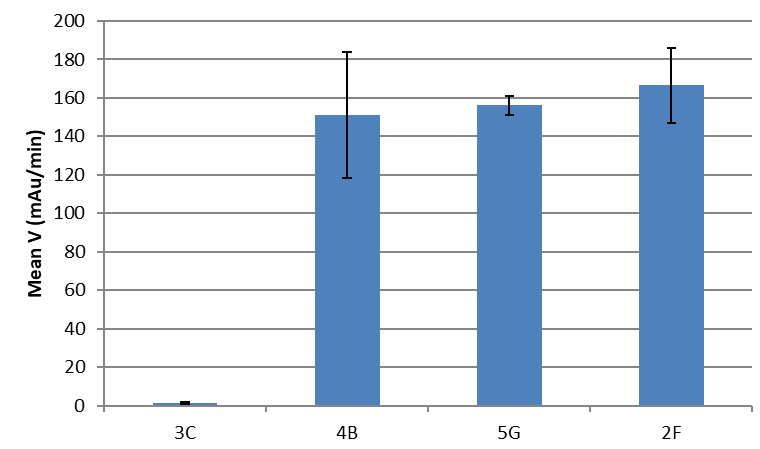


### MeHNL

#### Screening

##### Derepressed conditions


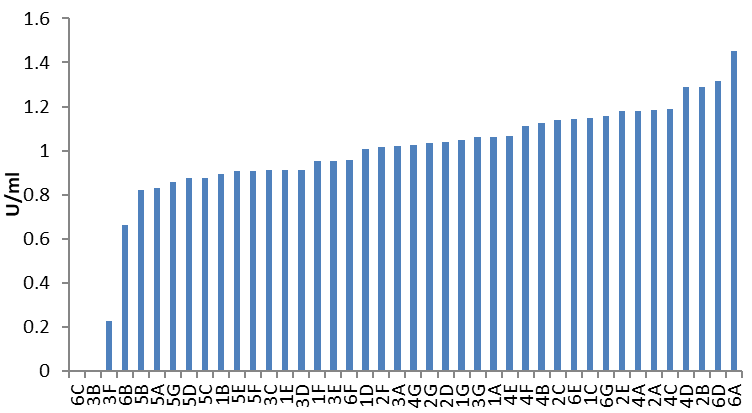


#### Rescreening

##### Methanol induction


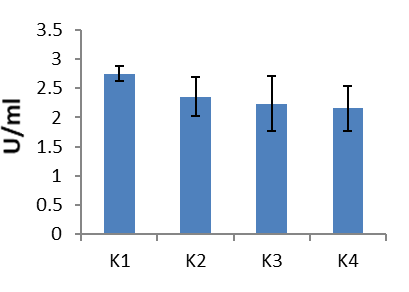


## S 4 – List of plasmids and strains used in this study

S 4: List of plasmids and strains used in this study.

| Reporter gene | Promoter | *P. pastoris* strain background (CBS 7435) | Reference |
| --- | --- | --- | --- |
| GFP | *HpFMD* | wildtype (mut+) | this study |
|  | *HpMOX* | wildtype (mut+) | this study |
|  | *CbFLD1* | wildtype (mut+) | this study |
|  | *CbAOD1* | wildtype (mut+) | this study |
|  | *PmMOD1* | wildtype (mut+) | this study |
|  | *PmMOD2* | wildtype (mut+) | this study |
|  | *PpAOX1* | wildtype (mut+) | (Vogl et al. 2016) |
|  | *PpCAT1* | wildtype (mut+) | (Vogl et al. 2016) |
|  | *PpGAP* | wildtype (mut+) | (Vogl et al. 2016) |
| HRP | *HpFMD* | mutS | this study |
|  | *PpAOX1* | mutS | (Vogl et al. 2016) |
|  | *PpCAT1* | mutS | (Vogl et al. 2016) |
| CalB | *HpFMD* | mutS | this study |
|  | *PpAOX1* | mutS | (Vogl et al. 2016) |
|  | *PpCAT1* | mutS | (Vogl et al. 2016) |
| MeHNL | *HpFMD* | wildtype (mut+) | this study |
|  | *PpAOX1* | wildtype (mut+) | this study |
|  | *PpCAT1* | wildtype (mut+) | this study |
